# Aggregate-selective antibody attenuates seeded aggregation but not spontaneously evolving disease in SOD1 ALS model mice

**DOI:** 10.1186/s40478-020-01032-2

**Published:** 2020-09-14

**Authors:** Manuela Lehmann, Matthew Marklund, Anna-Lena Bolender, Elaheh E. Bidhendi, Per Zetterström, Peter M. Andersen, Thomas Brännström, Stefan L. Marklund, Jonathan D. Gilthorpe, Ulrika Nordström

**Affiliations:** 1 Department of Clinical Science, Neurosciences, Umeå University, 901 87 Umeå, Sweden; 2 Department of Medical Biosciences, Pathology, Umeå University, 901 87 Umeå, Sweden; 3 Department of Medical Biosciences, Clinical Chemistry, Umeå University, 901 87 Umeå, Sweden; 4 Department of Integrative Medical Biology, Umeå University, 901 87 Umeå, Sweden

**Keywords:** ALS, SOD1, Template directed aggregation, Prion-like, Immunotherapy, Neurodegenerative disease

## Abstract

Increasing evidence suggests that propagation of the motor neuron disease amyotrophic lateral sclerosis (ALS) involves the pathogenic aggregation of disease-associated proteins that spread in a prion-like manner. We have identified two aggregate strains of human superoxide dismutase 1 (hSOD1) that arise in the CNS of transgenic mouse models of SOD1-mediated ALS. Both strains transmit template-directed aggregation and premature fatal paralysis when inoculated into the spinal cord of adult hSOD1 transgenic mice. This spread of pathogenic aggregation could be a potential target for immunotherapeutic intervention. Here we generated mouse monoclonal antibodies (mAbs) directed to exposed epitopes in hSOD1 aggregate strains and identified an aggregate selective mAb that targets the aa 143–153 C-terminal extremity of hSOD1 (αSOD1^143–153^). Both pre-incubation of seeds with αSOD1^143–153^ prior to inoculation, and weekly intraperitoneal (i.p.) administration attenuated transmission of pathogenic aggregation and prolonged the survival of seed-inoculated hSOD1^G85R^ Tg mice. In contrast, administration of a mAb targeting aa 65–72 (αSOD1^65–72^), which exhibits high affinity towards monomeric disordered hSOD1, had an adverse effect and aggravated seed induced premature ALS-like disease. Although the mAbs reached similar concentrations in CSF, only αSOD1^143–153^ was found in association with aggregated hSOD1 in spinal cord homogenates. Our results suggest that an aggregate-selective immunotherapeutic approach may suppress seeded transmission of pathogenic aggregation in ALS. However, long-term administration of αSOD1^143–153^ was unable to prolong the lifespan of non-inoculated hSOD1^G85R^ Tg mice. Thus, spontaneously initiated hSOD1 aggregation in spinal motor neurons may be poorly accessible to therapeutic antibodies.

**Electronic supplementary material:**

The online version of this article (10.1186/s40478-020-01032-2) contains supplementary material, which is available to authorized users.

## Introduction

ALS is characterized by a progressing adult-onset neuromuscular degeneration. The first symptoms typically present as locally restricted paresis that spreads contiguously and eventually results in generalized paralysis of most skeletal myotomes [11]. Death usually occurs within 1–3 years after first onset of paresis, as spreading paralysis engages the respiratory muscles [13]. Coding mutations in the *superoxide dismutase 1 (SOD1)* gene have been found in 2–6% of ALS patients with some variability among populations [1]. Most ALS-linked *SOD1* mutations destabilize the structure of the natively folded protein, resulting in an increased proportion of disordered and misfolded SOD1 species. Inclusions containing misfolded SOD1 in degenerating spinal motor neurons (MNs) are a hallmark of SOD1-mediated ALS in patients carrying *SOD1* mutations and in transgenic (Tg) models over-expressing mutant and/or human wild-type SOD1 (hSOD1^Wt^) [10, 19, 21, 42]. Multiple studies have also suggested increased levels of misfolded hSOD1^Wt^ in MNs and glial cells in sporadic ALS patients, and in patients carrying mutations in other ALS-linked genes [9, 14, 15, 25, 29, 34]. This suggests that hSOD1 aggregation may contribute to the pathogenesis and, thus, be a relevant therapeutic target for multiple subtypes of ALS.

To profile the structure of disease-relevant hSOD1 aggregates, we have developed a binary epitope mapping (BEM) technique. This is based on the dot blotting of aggregates trapped in a filter membrane, using an array of peptide antibodies that bind specifically to disordered hSOD1 sequences [6]. Using the BEM technique, we have identified two distinct SOD1 aggregate strains, denoted A and B, that can arise in the CNS of hSOD1 Tg ALS-model mice. Moreover, strain A or B aggregate seeds, prepared from Tg mouse or ALS patients spinal cord post mortem, transmit template-directed aggregation and induce premature fatal paralysis when inoculated into spinal cord of pre-symptomatic adult hSOD1 Tg mice [7, 8]. A number of other studies have provided additional evidence for a prion-like spread of hSOD1 aggregation in cell culture [5, 16, 17, 22, 27, 30], and in mouse models [2, 3, 5, 12, 16, 17, 27, 31, 32]. Together, these findings suggest that transmission of aggregation may be a key mechanism for the progressive spread of clinical signs and symptoms observed in ALS patients. Studies conducted in cell culture suggest that cell-to-cell transmission of pathogenic SOD1 aggregates involves their release into the extracellular space, for example via exocytosis or upon cell death, and that transmission can be reduced by pre-incubation of aggregate seeds with antibodies targeting hSOD1 [3, 17, 31]. This suggests that an immunotherapeutic approach that selectively targets hSOD1 aggregates may attenuate propagation of disease-associated aggregation within the CNS and, thereby, disease progression.

Here, we generated a set of monoclonal antibodies (mAbs) targeting disordered sequence elements that are accessible in hSOD1 aggregate strains isolated from the CNS of ALS model mice. We identified an aggregate-selective mAb that attenuated transmission of seeded aggregation and delayed premature fatal paralysis induced by inoculation of hSOD1 aggregate seeds in vivo. In contrast, long-term administration of the aggregate-selective mAb did not mitigate spontaneously evolving aggregation and ALS-like paralysis in non-inoculated hSOD1^G85R^ Tg mice.

## Results

### Development of hSOD1 aggregate-reactive monoclonal antibodies

**Fig. 1:**
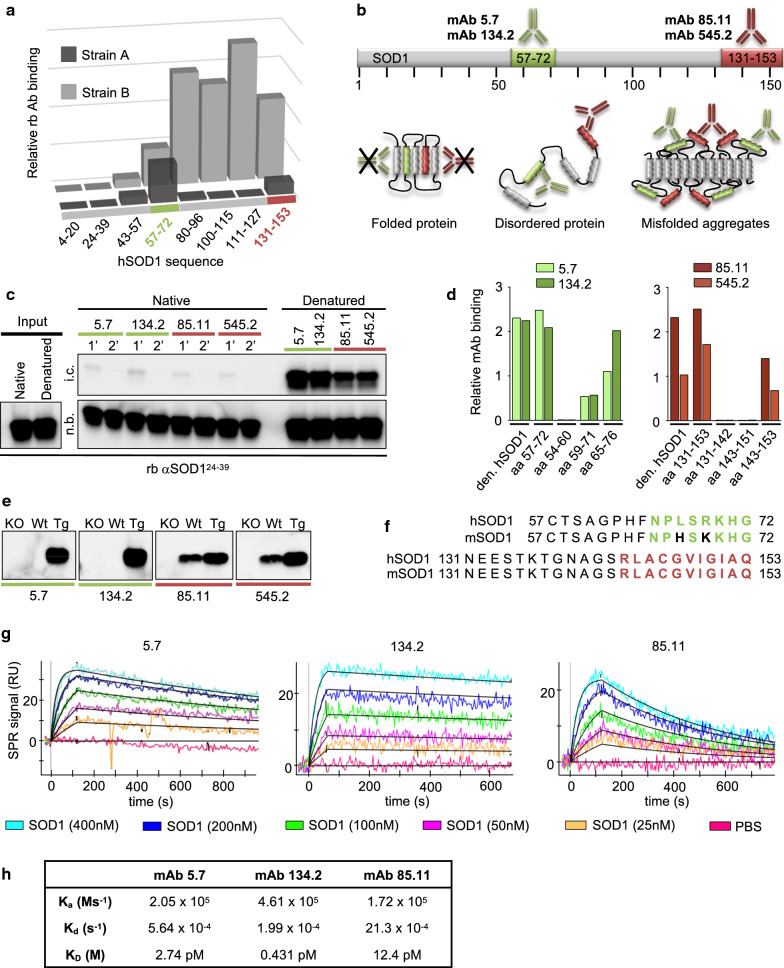
Mouse monoclonal hSOD1 antibodies specifically target disease associated hSOD1 species. **a** Graph represents binary epitope mapping patterns of strain A and strain B hSOD1 aggregates formed in vivo (modified from Bergh et al. [6]). Each bar represents the relative immunoreactivity of distinct epitopes of the hSOD1 protein sequence in dot blot assays of filter trapped hSOD1 aggregates from spinal cord homogenates. The aa 57–72 (green) and 131–153 (red) epitopes are available for binding in both A-, and B-aggregate strains, and were selected for the generation of aggregate reactive mouse monoclonal antibodies (mAbs). **b** Schematic representation of the hSOD1 protein sequence highlighting the epitopes used for immunisation. In the native protein, these epitopes are likely to be engaged in a rigidly folded structure and unable to adapt for antibody binding. In contrast, these epitopes are free and accessible for binding in disease-associated, unfolded/disordered, and aggregated hSOD1 species. **c** Immunoblots of immunocaptured native and denatured hSOD1 using mAbs immobilized on Dynabeads. To remove any traces of disordered hSOD1 in the native samples we performed two sequential incubations (1′ and 2′) of each sample. Input (1/200th of total input), non-bound (n.b., 1/200th of sample) and immunocaptured (i.c., 1/200th of sample) fractions were immunoblotted using rabbit antibody targeting aa 24–39 of hSOD1 (rbAb αSOD1^24–39^). **d** Graph showing relative reactivity of αSOD1 mAbs to full-length denatured hSOD1, or short peptides within the aa 57–72 (green bars) or aa 131–153 sequence (red bars) by ELISA. The target sequence of mAb clones 5.7 and 134.2 is within aa 65–72 and both mAb clones 85.11 and 545.2 target an epitope within the aa 143–153 sequence. **e** Western blots show mAb binding to murine and/or the Tg human SOD1 protein in spinal cord homogenates from C57BL/6J non-Tg control (Wt) and hSOD1^G85R^ Tg mice. SOD1 knockout (KO) spinal cord homogenate was included as negative control. See Additional file 1: Figure S1a for a full view of the filter. **f** The human SOD1 (hSOD1) peptide sequences used for immunization aligned to the murine SOD1 (mSOD1) epitope sequences. The specific mAb target sequences determined by the short peptide ELISAs are indicated in green and red. Non-conserved amino acids that distinguish murine, from the human sequence are highlighted in black. **g** SPR sensorgrams showing the binding of three antibodies towards different concentrations of denatured hSOD1. (h) Table summarizing the association rate (K_a_), dissociation rates (K_d_) and equilibrium dissociation constants (K_D_) of each mAb. K_a_ and K_d_ were calculated with the BIAevaluation software (Biacore AB) selecting *Fit kinetics simultaneous K*_*a*_*/K*_*d*_ (Global fitting) and 1:1 (Langmuir) interaction model. There was no data obtained from mAb 545.2, possibly due to low affinity. ms^−1^—millisecond, M—molar, s^−1^—second

Our previous results using BEM to profile disease-relevant hSOD1 aggregates suggest that they are composed of a repetitive structure containing both ordered and disordered segments [6, 7]. In strain A, two peptide sequences are inaccessible for binding by sequence-specific peptide antibodies (aa ~ 4 to 49 and ~ 100 to 127) (Fig. 1a). These segments are likely to be engaged in a rigidly packed fibrillar ‘core’ in aggregated hSOD1. Antibodies targeting peptide sequences in the intervening region (aa ~ 43 to 96) and the C-terminal tail of hSOD1 (aa ~ 131 to 153) bind strain A aggregates, suggesting that these sequences are exposed, disordered and may be available for immunotherapeutic targeting. In strain B, sequence elements from the N-terminal end to aa ~ 57 seem to be engaged in the fibrillar core, while the remaining sequence readily binds peptide antibodies against disordered hSOD1 sequence elements (Fig. 1a). Based on these patterns, we selected hSOD1^57–72^ and hSOD1^131–153^ as peptides to generate aggregate-reactive mouse mAbs (Fig. 1b).

### Monoclonal antibodies specific for disordered SOD1 sequences

Based on mAb reactivity to disordered sequences in monomeric or aggregated hSOD1 (Additional file 1: Table S1), we selected two mAb clones raised against each of the hSOD1^57–72^, and hSOD1^131–153^ peptides for further characterisation. We first performed immunocapture experiments to assess their specificity to disease associated disordered SOD1 species. The mAbs were coupled to magnetic beads and incubated with either native (holo-hSOD1) or disordered (denatured apo-hSOD1) protein at a 10-fold excess of the maximal binding capacity of each antibody. To trap trace amounts of disordered hSOD1 present in the native samples, we performed two sequential mAb incubations. All four mAbs captured minute amounts of hSOD1 in the first round, but no hSOD1 was detected in the second round of immunocapture (Fig. 1c). This is in line with our previous observations using rabbit (rb) Abs raised against the same hSOD1 peptide sequences [6]. Thus, all four mAbs were unable to bind to native hSOD1.

To define the target sequence of the candidate mAbs we performed ELISAs using short, overlapping peptides covering the sequences used for immunization (Fig. 1d). All four mAbs displayed similar binding to full-length denatured apo hSOD1 and the peptide used for immunization. By probing against shorter peptides, we narrowed down the target epitopes to include the aa 65–72 sequence and the aa 143–153 in the C-terminal extremity, respectively (Fig. 1d; Additional file 1: Table S1).

Next, we evaluated mAb specificity to hSOD1 in western blotting and immunohistochemistry assays. No reactivity was detected in samples from SOD1 knockout (SOD1 KO) mice, but all mAbs showed specific binding to a ~ 17 kDa protein representing hSOD1 in spinal cord homogenates from hSOD1^G85R^ Tg mice. The hSOD1^57–72^ mAbs showed no cross-reactivity with murine SOD1 (mSOD1) and were both specific for the hSOD1 protein (Fig. 1e and Additional file 1: Figure S1a). Since aa 67 and 69 differ between murine and hSOD1 (Fig. 1f), these data confirm the epitope mapping results and show that an epitope comprising residues L67 and/or R69 is important for binding. The sequence of SOD1^131–153^ is identical in hSOD1 and mSOD1, and the corresponding mAbs reacted equally with denatured SOD1 from both species (Fig. 1e and Additional file 1: Figure S1a). In summary, these mAbs target epitopes that are specific for non-native hSOD1 species harbouring disordered sequences comprising hSOD1^65–72^ and hSOD1^143–153^, respectively. Finally, we determined the binding affinities of immobilized mAbs to denatured hSOD1 using surface plasmon resonance (SPR). The mAb 134.2 exhibited the lowest equilibrium dissociation constant (K_D_ = K_d_/K_a_ = 0.431 pM) indicating the strongest binding to monomeric disordered hSOD1. In contrast, the mAb 85.11 displayed a high dissociation constant (K_D_ = 12.4 pM) and a high dissociation rate (K_d_ = 21.3 × 10^−4^) implying low affinity and rapid separation from monomeric disordered hSOD1 (Fig. 1g, h).

### Verification of mAb binding to spinal cord-derived hSOD1 aggregates

**Fig. 2:**
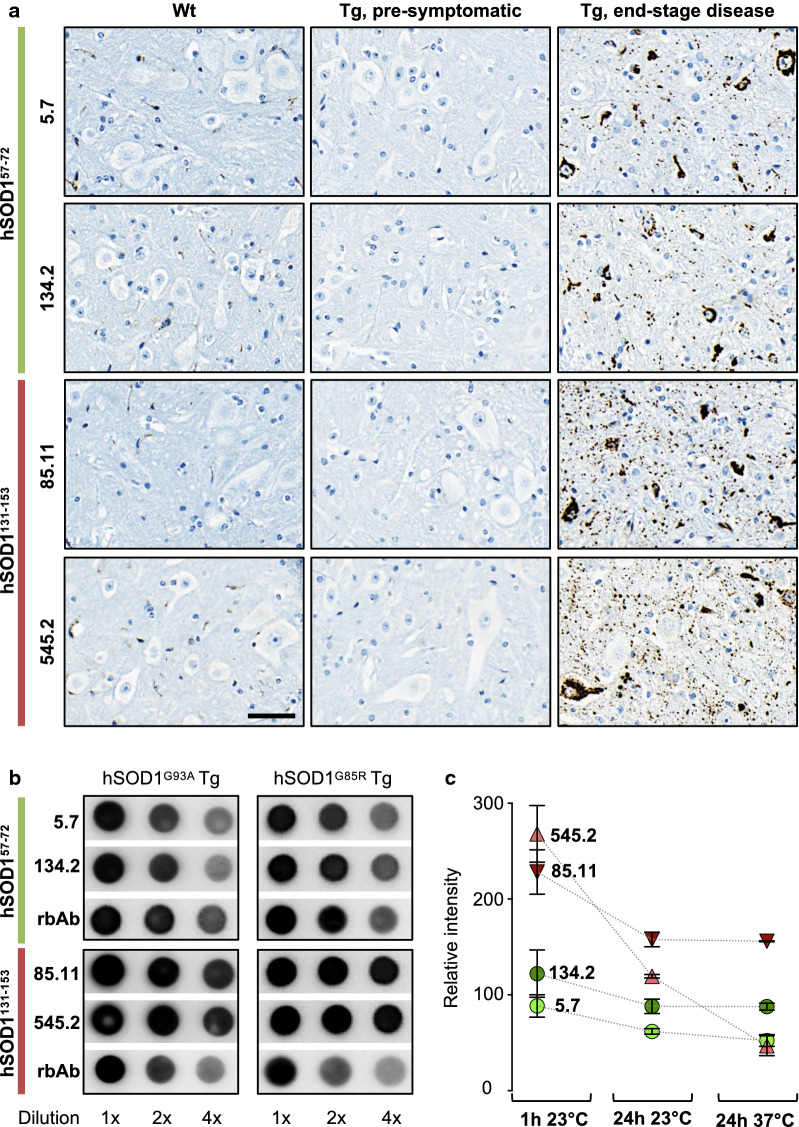
Mouse monoclonal antibodies bind spinal cord derived hSOD1 aggregates. **a** Immunohistochemical staining of proteinase-resistant hSOD1 aggregates in the ventral horn of spinal cord sections from C57BL/6J non-Tg control (Wt); 200-day-old pre-symptomatic hSOD1^G85R^ Tg; and 400-day terminal stage Tg hSOD1^G85R^ mice, using mAbs raised against the aa 57–72 peptide (hSOD1^57–72^) or aa 131–153 (hSOD1^131–153^). Scale bar = 40 μm. **b** Dot blot of filter-captured aggregates from spinal cords of end-stage disease hSOD1^G85R^ and hSOD1^G93A^ Tg mice, probed with the indicated αSOD1 mAbs. Polyclonal rabbit antibodies raised against the same peptides (rbAb hSOD1^57–72^; rbAb hSOD1^131–153^) were included as positive controls. **c** Graph represents relative staining intensities of the hSOD1^G85R^ aggregates. To assess aggregate/mAb complex stability, filters were either washed for 1 h at 23 °C or exposed to extended 24 h washing at 23 °C or 37 °C (n = 3 for each condition). Data presented as mean ± SD

To analyse mAb binding to disease-relevant hSOD1 aggregates in the CNS, we developed an immunohistochemistry protocol that exploited the use of restricted proteolysis in tissue sections. This resulted in preferential digestion of diffusely distributed monomeric and oligomeric species but hSOD1 aggregate reactivity remained largely intact. After proteinase treatment, no staining was detected in SOD1 KO, non-Tg control (Wt) or pre-symptomatic SOD1^G85R^ Tg spinal cord (Fig. 2a), which contain disordered monomeric hSOD1 but no detectable aggregation [7] (Additional file 1: Figure S1b). However, all four mAbs labelled distinct aggregates in spinal cord tissue from paralytic, end-stage SOD1^G85R^ Tg mice. Hence, all four mAbs bind in vivo relevant hSOD1 aggregates.

We also investigated mAb aggregate reactivity in homogenates of fresh-frozen spinal cord from terminal stage hSOD1^G85R^ or hSOD1^G93A^ Tg mice. Dot blot analysis showed that the hSOD1^143–153^ mAbs exhibited higher reactivitiy to filter trapped hSOD1 aggregates compared to the hSOD1^65–72^ mAbs (Fig. 2b). Extended washing indicated that clone 85.11 (αSOD1^143–153^) formed the strongest binding complex with hSOD1 aggregates (Fig. 2c). The combined results from our mAb characterization suggest that this antibody exhibits a high dissociation constant for monomeric disordered hSOD1 (Additional file 1: Table S1), but strong reactivity and avidity towards aggregates. Due to this aggregate selectivity, we chose the αSOD1^143–153^ for a proof of principle study aimed at blocking transmission of seeded aggregation in vivo.

### An aggregate-selective αSOD1^143–153^ mAb attenuates seeding of hSOD1 aggregation in vivo

**Fig. 3:**
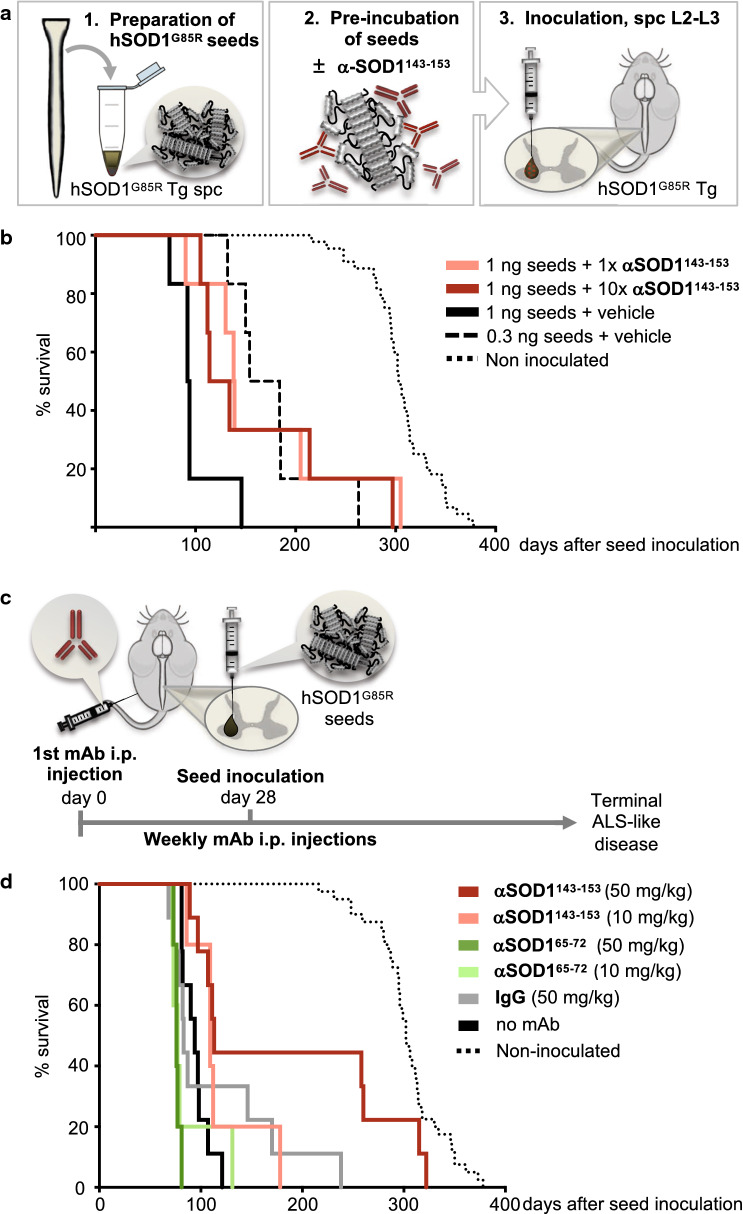
αSOD1^143–153^ prolongs survival of hSOD1 seed inoculated hSOD1^G85R^ Tg mice. **a** Overview of experimental protocol in the hSOD1-inoculation mouse model. Aggregate seeds prepared from terminal stage hSOD1^G85R^ Tg spinal cords were incubated with mAb αSOD1^143–153^ or vehicle control prior to seed inoculation into the lumbar spinal cord (L2–L3 level) of pre-symptomatic Tg hSOD1^G85R^ mice. **b** Kaplan–Meier plot show post-inoculation survival of hSOD1^G85R^ mice. Seeds and mAbs were incubated at equal molar ratio (1 ng seeds + 1× αSOD1^143–153^) (dark red line; n = 6) or 10-times the molar ratio of mAb (1 ng seeds + 10× αSOD1^143–153^) (light red; n = 6). Analyzed as one group, mice inoculated with seeds mixed with αSOD1^143–153^ (n = 6 + 6) had a significantly longer post-inoculation survival time compared to mice inoculated with seeds mixed with PBS vehicle, (1 ng seeds + vehicle) (black line; n = 6; *p* < 0.05). For dilution effect comparison, another group of mice were inoculated with diluted seeds (0.33 ng seeds + vehicle) (dashed black line; n = 6). The total injection volume in each mouse was 1 μL of the respective preparation. Survival time of non-inoculated control hSOD1^G85R^ Tg mice from the same colony is included for comparison (dotted black line; n = 44). Non-inoculated control values are presented as age of sacrifice minus mean inoculation age (107 days) for the treated groups. **c** Scheme for mAb injection in the hSOD1^G85R^ seed inoculation mouse model: Once weekly systemic administration of mAbs via i.p injection (day 0), was initiated 3 weeks before hSOD1^G85R^ aggregate seeds were inoculated into the lumbar spinal cord (day 28, one day after the 4th mAb injection). Antibodies were injected i.p. once per week until the mouse reached terminal disease stage. **d** Kaplan–Meier plot showing survival in days post hSOD1^G85R^ aggregate seed inoculation. Treatments using 10 mg/kg αSOD1^65–72^ (light green; n = 5), 10 mg/kg αSOD1^143–153^ (light red; n = 5) or 50 mg/kg control IgG (grey; n = 9) had no significant effect on survival compared to no mAb control treatment (black; n = 9). Treatment with 50 mg/kg αSOD1^65–72^ (green; n = 5) significantly decreased survival (see Table 1). Treatment with 50 mg/kg αSOD1^143–153^ (red; n = 9) significantly extended survival. For comparison, survival of non-inoculated control hSOD1^G85R^ transgenic mice is shown as black dotted line (n = 44) and represents age at end-stage disease minus 100-days, the mean age at seed inoculation

To test if αSOD1^143–153^ could block the induction and course of seeded aggregation in vivo, we used a previously described model based on the hSOD1^G85R^ Tg mouse inoculated with strain A seeds [7]. This triggers a templated prion-like spread of hSOD1 aggregation that is highly reproducible. Seeds were prepared from the spinal cord of end-stage Tg hSOD1^G85R^ mice (see Methods) and a preparation containing ~ 1 ng/μL hSOD1 aggregates was incubated with αSOD1^143–153^ at a stoichiometric ratio of 1:1 or 1:10 (monomeric hSOD1:mAb), or with endotoxin-free PBS as a vehicle control. One μl of the respective, preparations were then inoculated into the left ventral horn of the lumbar spinal cord of pre-symptomatic adult (~ 100-day-old) mice (Fig. 3a). Pre-incubation of seeds with αSOD1^143–153^ resulted in a mean prolongation of survival time by 69 days (35%) for a 1:1 ratio and 64 days (32%) for a 1:10 ratio, compared to mice inoculated with seeds incubated with vehicle control (Fig. 3b; Additional file 1: Table S2). This suggests that αSOD1^143–153^ bound to and incapacitated a proportion of the infectious particles in the inoculate, similar to a dilution effect. Consistently, inoculation of seeds diluted to a three-fold lower amount (0.33 ng hSOD1/μl) prolonged survival by 79 days (40%) compared to non-diluted (1 ng/μl) seeds (Fig. 3b). These proof of principle results encouraged us to test the efficacy of systemically administered mAbs to block seeded transmission of hSOD1 aggregation in vivo.

### Systemic administration of α-SOD1^131–153^ attenuates transmission of seeded aggregation in the CNS

We first assessed basic pharmacokinetic parameters including elimination rate and distribution after a single i.p. injection of 50 mg/kg mAb in Wt mice. The mAb αSOD1^65–72^ (clone 134.2), having the strongest binding for disordered, monomeric hSOD1 (Fig. 1g, h; Additional file 1: Table S1) was used as a “monomeric-selective” control. Both αSOD1^65–72^ and αSOD1^143–153^ showed a typical pharmacokinetic profile in plasma with rapid distribution and slow elimination (Additional file 1: Figure S2a). The half-life for both mAbs was > 7 days suggesting that they were maintained in circulation and that administration via weekly i.p. injection would be sufficient to assess in vivo efficacy.

To assess whether peripherally administered mAbs could attenuate seeded SOD1 aggregation in the spinal cord, we treated mice with once weekly i.p injections of αSOD1^143–153^ (IgG1) or αSOD1^65–72^ (IgG2b) or purified polyclonal mouse IgG, which was included as a control for both αSOD1 isotypes. Weekly mAb administration started 3 weeks before spinal cord inoculation of hSOD1^G85R^ seeds and was continued until animals reached a paralytic end-stage (Fig. 3c). Treatment with 50 mg/kg/week of αSOD1^143–153^ significantly prolonged mean survival by an average of 91 days (47%) compared to a control group of untreated (no mAb control) mice. A lower dose (10 mg/kg/week), prolonged the mean survival by 24 days (12%). In contrast, αSOD1^65–72^ (50 mg/kg/week) treatment resulted in a significantly shorter mean survival (-18 days, -9%) (Fig. 3d; Table 1). There was no significant difference in survival in the 50 mg/kg polyclonal mouse IgG treated control group compared to the no mAb control group. Thus, peripherally administered αSOD1^65–72^ with high affinity for monomeric SOD1 had a significant adverse effect on lifespan in this model. In contrast, the aggregate-selective αSOD1^143–153^ mAb attenuated seeded hSOD1 aggregation in the spinal cord.

**Table 1:** Disease and survival data of seed inoculated hSOD1^G85R^ Tg mice receiving systemic administration of mAbs

	No mAb control		αSOD1^65–72^ (10 mg/kg)		αSOD1^65–72^ (50 mg/kg)		αSOD1^143–153^ (10 mg/kg)		αSOD1^143–153^ (50 mg/kg)		pAb IgG (50 mg/kg)	
	n	days	n	days	n	days	n	days	n	days	n	days
Age at 1st mAb injection	–	–	5	76 ± 2	5	76 ± 2	5	79	9	79 ± 3	9	78 ± 3
Age at SOD1^G85R^ seed inoculation	9	100 ± 4	5	98 ± 2	5	99 ± 2	5	101 ± 1	9	101 ± 3	9	100 ± 3
Paralytic end-stage (days post-inoculation)	9	94 ± 14	5	88 ± 24	5	78 ± 2	5	118 ± 35	9	185 ± 101	9	113 ± 59
Symptom onset (days post-inoculation)	6	69 ± 13	5	73 ± 17	5	62 ± 5	5	100 ± 25	9	175 ± 102	9	101 ± 63
Age at paralytic end-stage	9	195 ± 13	5	187 ± 26	5	177 ± 3**	5	219 ± 35	9	286 ± 100**^,^ *	9	214 ± 58

For experimental outline and survival graph; see Fig. 3c, d

All data are presented as mean ± SD

– not applicable

*p < 0.05 survival is significantly different compared to IgG control

**p < 0.01 survival is significantly different compared to No mAb control

### αSOD1^143–153^ associates with hSOD1 aggregates in vivo

**Fig. 4:**
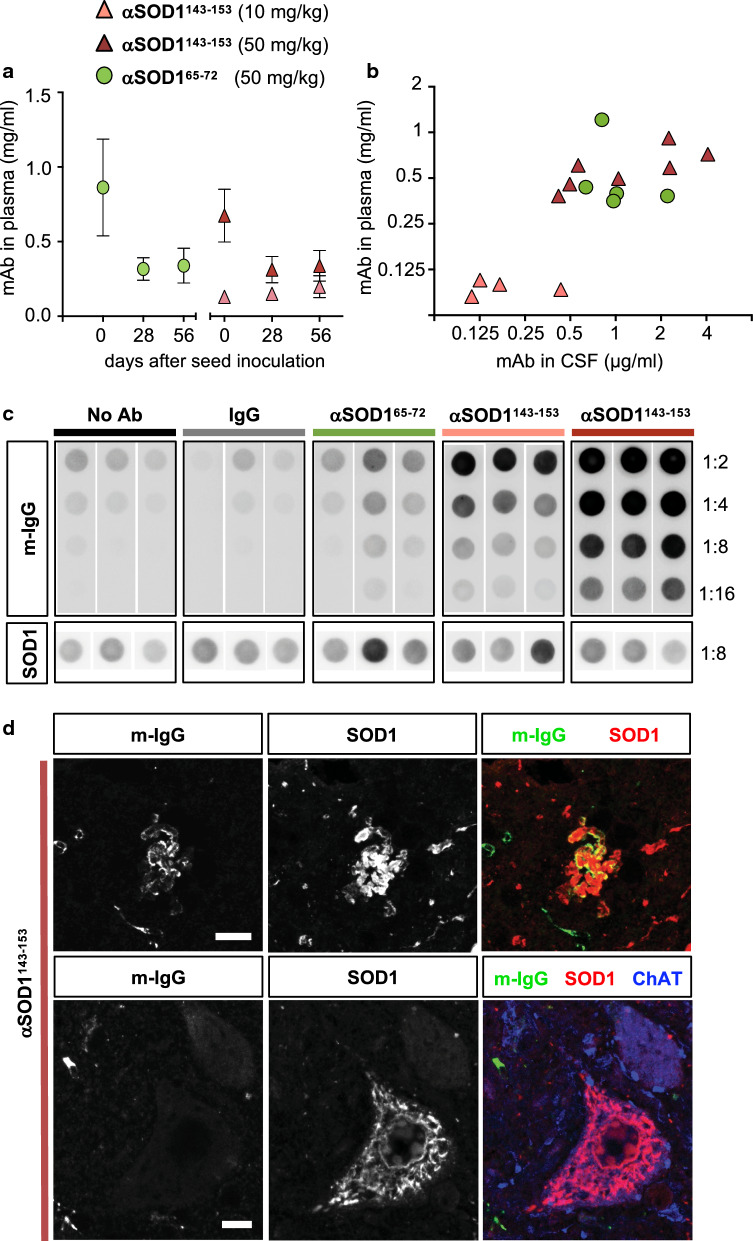
αSOD1^143–153^ detected in association to hSOD1 aggregates formed in the CNS. **a**, **b** Plots show antibody concentrations in plasma and CSF samples from hSOD1^G85R^ Tg mice inoculated with aggregate seeds and treated with weekly i.p. mAb injections; 10 mg/kg αSOD1^143–153^ (pink triangles), 50 mg/kg αSOD1^143–153^ (red triangles), 50 mg/kg αSOD1^65–72^ (green circles). αSOD1 mAb titres were determined by hSOD1 ELISA. **a** Blood samples were collected on the day of seed inoculation (d0); 1 day after the latest antibody injection, day 28 and day 56 after seed inoculation (d28, d56); 7 days after antibody injection. Values represent mean ± SD (n = 4–5 mice/group for each timepoint). **b** Scatter plot shows αSOD1 mAb concentration in CSF in relation to plasma level. Days post mAb injection varied depending on when the end-stage phenotype was reached. Both blood and CSF samples were collected at sacrifice. Mean CSF concentration was calculated to 0.3 μg/ml, 0.22% of plasma levels (10 mg/kg αSOD1^143–153^ (n = 4), 1.6 μg/ml, 0.25% of plasma levels in the 50 mg/kg αSOD1^143–153^ mice (n = 7) and 1.1 μg/ml, 0.26% of plasma levels in 50 mg/kg αSOD1^65–72^ treated mice (n = 5). **c** Dot blot of cervical spinal cord homogenates from terminal stage non-, or mAb-treated mice probed with anti-mouse IgG (m-IgG) to monitor mAb binding in association to filter trapped aggregates (n = 3 mice/group). As control for aggregate load, the level of hSOD1 aggregates in each homogenate, was monitored using a polyclonal rbAb SOD1^57–72^ (for quantification see Additional file 1: Figure S1). **d** Confocal images of the ventral horn of cervical spinal cord sections from αSOD1^143–153^ treated mice. Immunofluorescence labelling using anti-mouse IgG (m-IgG; green), SOD1 (red) and ChAT (blue) as a marker for MNs. Scalebars = 10 μm. In only a few occurrences, could IgG labelling be detected in association to hSOD1 aggregation in αSOD1^143–153^ treated animals. No IgG labelling was detected in association with hSOD1 aggregates in ChAT^+^ motor neurons

We next analysed mAb titres in samples of blood and CSF to assess if differences in circulating mAb titres could explain the differences in outcome between the two different αSOD1 treatment groups. ELISA analysis confirmed slow elimination and steady mAb titres in plasma over the course of the immunotherapy study (Fig. 4a). CSF samples from the cisterna magna and the last blood samples were withdrawn during euthanization. End-stage was decided independently of the number of days that had passed since the last i.p. injection. Consequently, terminal plasma mAb titres varied more than at earlier, synchronized time points. However, αSOD1 titres detected in plasma and CSF samples from both αSOD1^143–153^, and the αSOD1^65–72^ treated mice reached similar levels, with CSF mAb levels reaching 0.1–0.6% of the plasma titre (Fig. 4b).

Next, we investigated whether mAbs could be detected in association with aggregates in the CNS of treated mice. Circulating mAbs were washed out using transcardial perfusion with PBS before spinal cord tissue isolation. Aggregates from cervical spinal cord homogenates were captured on dot blot filters and probed with rabbit anti-hSOD1 or anti-mouse IgG. The levels of aggregated hSOD1^G85R^ were similar in cervical spinal cord homogenates from no mAb controls and mAb treated mice (Fig. 4c; Additional file 1: Figure S2d). Marked anti-mouse IgG immunoreactivity was detected with filter trapped aggregates isolated from αSOD1^143–153^ treated mice. Stronger signal was detected in animals treated with the higher 50 mg/kg dose compared to the 10 mg/kg group, suggesting a dose dependent binding of the αSOD1^143–153^ to aggregates in the spinal cord (Fig. 4c; Additional file 1: Figure S3a). This was in contrast to spinal cord homogenates from the αSOD1^65–72^ treated, the polyclonal mouse IgG treated, and the no mAb control group, which all exhibited similarly weak anti-mouse IgG reactivity. Western blot analysis of spinal cord homogenates showed that total hSOD1 and mSOD1 levels were unaltered in treated animals (Additional file 1: Figure S2b, c). In summary, both αSOD1^65–72^ and αSOD1^143–153^ reached comparable circulating levels in plasma and CSF and both soluble and aggregated hSOD1 levels were similar in all treatment groups. Nonetheless, we only detected robust mouse IgG labelling in filter trapped aggregates from spinal cords of mice treated with the aggregate-selective αSOD1^143–153^.

### Aggregate conformation is unaltered in αSOD1^143–153^ treated mice

Different strains of hSOD1 aggregates display differences in templated growth kinetics [7]. This means that differences in strain characteristics can affect time of incubation to symptom onset, disease phenotype and survival time [6, 7]. We used BEM to investigate whether the presence of sequence specific mAbs affected aggregate conformation in end-stage animals. Aggregates from both αSOD1^65–72^ and αSOD1^143–153^ treated mice displayed a typical strain A pattern (Additional file 1: Figure S2e). Therefore, we conclude that neither the lack of αSOD1^65–72^ binding in vivo, nor the prolonged survival of αSOD1^143–153^ treated mice, can be attributed to the appearance of strains with different fibril structures.

### Distribution of hSOD1^G85R^ pathology is unaltered in treated mice

To investigate if mAb treatment altered distribution of hSOD1 pathology, or MN degeneration, we performed immunohistopathological analysis of tissue sections from cervical spinal cord of end-stage mice. When compared to pre-symptomatic 200-day-old hSOD1^G85R^ Tg mice, we found a significant loss of choline acetyltransferase (ChAT) labelled MNs of a large (> 300 mm^2^), but not of a smaller (< 300 mm^2^), diameter. However, there was no difference between treatment groups (Additional file 1: Figure S3c and d). We also examined co-localization of hSOD1 aggregates and the astrocyte marker glial fibrillary acidic protein (GFAP), or the microglia marker ionized calcium binding adaptor molecule 1 (Iba1). We could only detect a few rare instances where punctate hSOD1 labelling colocalized with GFAP^+^ or Iba1^+^ cells (Additional file 1: Figure S3b and S4). Instead, the major proportion of hSOD1 aggregation was localized to the neuropil, or in ChAT^+^ MNs (Fig. 4d; Additional file 1: Figure S3a) as previously reported for the hSOD1 prion mouse model [8]. This distribution remained unaltered, independent of mAb treatment.

We next performed co-labelling of anti-mouse IgG and anti-hSOD1 in an attempt to visualize any αSOD1^143–153^ associated with hSOD1 aggregates. We detected anti-mouse IgG labelling localized with hSOD1 aggregate assemblies in the neuropil (Fig. 4d; Additional file 1: Figure S3a), and possibly the remnants of dead cells surrounded by Iba1^+^ microglial rosettes (Additional file 1: Figure S3b). However, we were not able to detect any anti-IgG labelling of the numerous hSOD1 aggregates within ChAT^+^ MNs (Fig. 4d; Additional file 1: Figure S3a). This may indicate that αSOD1^143–153^ binds to hSOD1 aggregates in the extracellular space but has limited access to intracellular aggregates.

The finding of microglial rosettes surrounding αSOD1^143–153^ labelled aggregates prompted us to assess microglial activation by quantification of Iba1 immunofluorescence staining in cervical spinal cord sections. There was a significant increase in the number of Iba1^+^ cells in the ventral horn at end-stage, compared to pre-symptomatic 200-day-old hSOD1^G85R^ Tg mice, but no difference between treatment groups (Additional file 1: Figure S4b and c). In summary, we found no evidence for altered glial pathology in αSOD1^143–153^ treated mice.

### Long-term α-SOD1^143–153^ immunotherapy does not prolong lifespan of non-seeded Tg mice

**Fig. 5:**
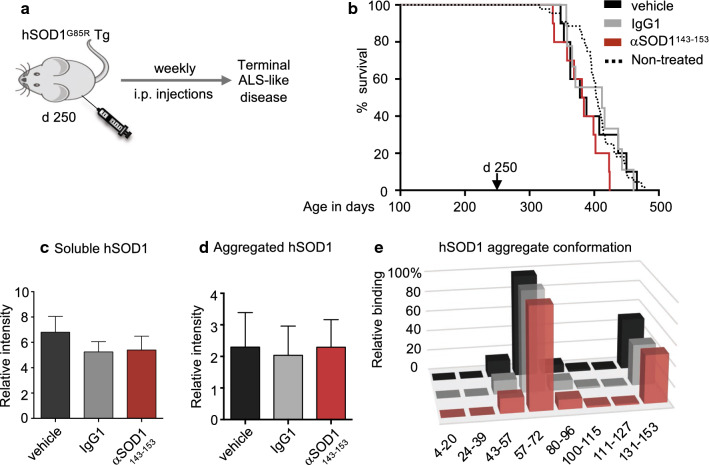
αSOD1^143–153^ does not attenuate spontaneous aggregation and ALS-like disease in hSOD1^G85R^ Tg mice. **a** Outline of the mAb injection scheme in the hSOD1^G85R^ Tg mouse model (non-seed inoculated mice): once weekly peripheral administration of mAbs via i.p injections was initiated before accumulation of detectable SOD1 aggregation (d250) and continued until mice reached the paralytic end-stage. **b** Kaplan–Meier plot showing survival in days. There was no significant difference between curves (n = 9–10 animals/group). Survival of non-treated hSOD1^G85R^ Tg mice included for comparison (black dotted line). See also supplementary table S3. Graphs represent: **c** level of soluble hSOD1 in cervical spinal cord samples determined by western blot. Values were normalized to total protein in the samples determined by BCA assay. mAb treatment did not result in significantly lowered levels of soluble hSOD1 (n = 6 animals/group). **d** Level of aggregated hSOD1 in cervical spinal cord analyzed by dot blot using rbAb SOD1^57–72^. mAb treatment had no significant effect on aggregate load in terminal stage in hSOD1^G85R^ Tg mice (n = 6 animals/group). **e** Conformation of hSOD1 aggregates in spinal cord samples determined by binary epitope mapping. Bars represent the mean reactivity of the 8 peptide antibodies used to probe filter trapped spinal cord derived aggregates from the respective treatment group: Vehicle treatment (black): 50 mg/kg αSOD1^143–153^ (red); 50 mg/kg IgG1 (grey) (n = 6 animals/group). Relative binding to the hSOD1 sequences is presented as the percentage of rbAb SOD1^57–72^ binding (set as 100%). mAb treatment did not alter the conformation of strain A aggregates forming in the hSOD1^G85R^ Tg spinal cord

Since αSOD1^143–153^ treatment prolonged survival in seed inoculated hSOD1^G85R^ Tg mice, we next studied the efficacy of long-term therapy in non-seeded mice. We used the same regimen of weekly i.p. injection with the 50 mg/kg dose but started at 250-days-of-age, which is approximately 100 days before the mean age of symptom onset and 150 days before the expected paralytic end-stage (397 ± 49 days) (Fig. 5a). There was no significant difference in mean survival between mice injected with αSOD1^143–153^, isotype control mAb (IgG1); or vehicle control (PBS) (Fig. 5b).

Western blot analysis of spinal cord homogenates showed that levels of both total and aggregated hSOD1 were unaltered in treated animals (Fig. 5c, d). BEM assay revealed that aggregates from spinal cords of end-stage αSOD1^143–153^, IgG1, and vehicle treated mice displayed a typical strain A pattern (Fig. 5e). Therefore, we conclude that the lack of efficacy in non-inoculated hSOD1^G85R^ Tg mice is not due to formation of an alternative hSOD1 aggregate strain that escapes α-SOD1^131–153^ mAb binding. In conclusion, the aggregate-selective αSOD1^143–153^ attenuated premature disease transmitted by seeding. Nonetheless, it was unable to suppress spontaneous hSOD1 aggregation induced by transgenic overexpression in ageing hSOD1^G85R^ Tg mice.

## Discussion

We show that mAb targeting aa 143–153 in the C-terminal extremity of hSOD1 attenuated transmission of seeded hSOD1 aggregation in vivo. The αSOD1^143–153^ mAb exhibited low affinity and a large dissociation constant for monomeric disordered hSOD1. Nonetheless, it displayed strong reactivity and retained complex stability to aggregated hSOD1 in dot blot assays. This implies that the aggregate-selective nature of αSOD1^143–153^ depends on avidity, where C-terminal tails protruding from the fibrillar core of hSOD1 aggregates are close enough to enable bivalent mAb binding (see Fig. 1b). In contrast, αSOD1^65–72^ was unable to attenuate seeded transmission in vivo and was not associated with aggregates isolated from treated mice. Both mAbs reached similar levels in the CSF and, although αSOD1^65–72^ had lower reactivity with hSOD1 aggregates, it outperformed αSOD1^143–153^ in all other in vitro assays for hSOD1 reactivity.

The aa 65–72 epitope is part of an exposed disordered loop in aggregated hSOD1^G85R^. The loop structure may partially restrict adaptation of the aa 65–72 sequence to the antigen-binding site of αSOD1^65–72^, or limit access for bivalent antibody binding. In combination with the high affinity to monomeric apo-SOD1, this might promote preferential binding to more flexible, soluble disordered SOD1 species and, hence, sequestration of the antibodies. Such off-target effects might contribute to the lack of efficacy seen with the αSOD1^65–72^. The reason for shorter survival in the αSOD1^65–72^ treatment group is unclear. Possibly immunotherapy targeting certain epitopes of disordered hSOD1 species might enhance pathogenic interactions and accelerate disease progression. Sábado et al. have previously reported an adverse effect of an active immunotherapeutic approach targeting the aa 29–57 in hSOD1 Tg mice [35]. Similarly, Wood et al. demonstrated that chronic wasting disease in elk was accelerated by active vaccination with a linear unfolded prion protein epitope [43].

Our results suggest that αSOD1^143–153^ can engage with hSOD1 aggregates in vivo. We performed immunohistochemical analyses with the aim of visualizing mAb-SOD1 binding in tissue sections. Still, we only detected occasional co-localization of anti-mouse lgG labelling and hSOD1 aggregates, mainly in the interstitial space or associated with microglia, possibly indicating microglia phagocytosis of mAb-bound aggregates.

Although we confirmed the presence of extensive aggregation within MNs, we did not detect mouse IgG in association with hSOD1 labelling in ChAT^+^ MNs. This implies that peripherally administered mAbs have poor access to intracellular aggregates. Moreover, preincubation of αSOD1^143–153^ was sufficient to attenuate seeding of templated aggregation, suggesting that mAb binding to extracellular aggregates is sufficient to block transmission of templated aggregation induced by inoculation of aggregate seeds. Hence, therapeutic approaches attempting to reduce spreading aggregation based on anti-hSOD1 mAbs may only be effective if aggregate transmission involves an exposed, extracellular route, such as on the surface of extracellular vesicles, or as naked aggregates in the interstitial space following cell death [17, 38].

In our experiments, spontaneously initiated aggregation driven by Tg hSOD1^G85R^ overexpression was apparently inaccessible to antibody therapy, as long-term systemic administration of αSOD1^143–153^ did not alter the level of aggregated hSOD1 or survival time in the hSOD1^G85R^ Tg mouse model. These results are in line with previous reports from either active immunization or passive antibody-based approaches in high copy number Tg models of SOD1-mediated ALS. Most studies have reported modest therapeutic efficacy, with a few days prolonged survival, or even adverse effects [18, 23, 35, 39, 40]. Apart from poor access to intracellular aggregation, another explanation for lack of effect on spontaneous disease in the current study might be an insufficient antibody titre in the CNS. Intrathecal or intracerebroventricular infusions would result in higher antibody titres and potentially increase the ratio of mAb taken up by motor neurons and binding to pathogenic hSOD1 species in the CNS. Indeed, previous studies have shown a modest but significant effect, even in the aggressive short-lived hSOD1^G93A^ G1H model [18, 25]. However, due to technical challenges with long-term treatment, we did not test this route of administration.

Immunotherapeutic approaches targeting misfolded hSOD1 have only resulted in substantially prolonged survival when tested in the slowly progressing hSOD1^G37R^ Tg mouse model [18, 23, 25, 44]. In these mice, both active and passive immunotherapy resulted in 40 and 59 days of increased survival compared to vehicle control, respectively. The hSOD1^G37R^ and the hSOD1^G85R^ Tg mice have comparable survival times (Fig. 5; Additional file 1: Table S3 and [42]). Hence, a critical factor in study designs may be the age at which the treatments were initiated. The studies in the hSOD1^G37R^ Tg model were initiated at 45 and 90 days-of-age, compared to injections starting at 250 days in the present study. Future studies targeting misfolded species in the hSOD1^G85R^ Tg model should consider initiating treatment at an earlier stage.

Liu et al. have used an active immunotherapeutic approach, based on vaccination with a peptide corresponding to the C-terminal aa 143–151 of hSOD1 [23, 34]. Maier et al. used an antibody targeting aa 76–82 of hSOD1 [25]. Although the epitope availability of hSOD1^G37R^ aggregates has yet to be established, both strategies target sequences that are; i) available for binding in strain A and B aggregates and ii) closely associated to the sequences targeted by the αSOD1^143–153^ and αSOD1^65–72^ (Fig. 1a) [6]. Both studies reported attenuated accumulation of oligomeric [23], aggregated [25], as well as monomeric hSOD1 species in spinal cord tissue of treated mice.

Previous studies have shown that the isotype-specific Fc domain and its effector functions can influence the degree of aggregate clearance and neuronal protection in ALS and other models of neurodegenerative diseases [4, 20, 25]. Hence, differences in the mAb isotypes used; IgG2a (α-miSOD1^(76–82)^ [25]); IgG1 (αSOD1^143–153^) and IgG2b (αSOD1^65–72^), might be responsible for the lack of reduction of misfolded hSOD1 species reported here. Future studies should consider using an engineered mAb carrying the CDR region of the aggregate selective αSOD1^143–153^ in combination with the IgG2a Fc-domain, proven to be effective in immunotherapy aiming to deplete accumulation of disease associated proteins [25, 28, 37, 41].

In conclusion, our results suggest that mAbs exhibiting aggregate selectivity, based on low affinity for monomeric disordered hSOD1 species and high avidity for aggregated protein, can reduce transmission of templated aggregation in vivo. However, targeting neoantigen epitopes present only in the aggregate structure might be a more effective approach, since this could achieve high aggregate specificity whilst minimizing the risk of off-target interactions with soluble disordered hSOD1 species. Developing new methods to isolate and amplify pure hSOD1 aggregates with disease-relevant structures could be an important step for future generation of improved hSOD1 aggregate specific mAbs.

## Materials and methods

### Reagents and chemicals

Reagents and chemicals were obtained from Sigma or Thermo Fisher Scientific unless stated otherwise.

### Generation of mouse monoclonal antibodies

Synthetic peptides corresponding to aa 57–72 and aa 131–153 of the SOD1 sequence were injected into female BALB/c mice. B-cells were isolated and fused to SP 2/0 myeloma cells. Immortalized hybridoma cells were sub-cloned and maintained in Dulbecco’s Modified Eagle Medium containing 4.5 g/l glucose and 4 mM l-glutamine. Media was supplemented with 10% (v/v) foetal bovine serum (FBS), 1 mM sodium pyruvate, and penicillin/streptomycin. Cells were incubated at 37 °C in a humidified atmosphere supplemented with 5% (v/v) CO_2_. Initial screening for antigen binding was performed using standard ELISA techniques using disordered apo-hSOD1 (see below).

For large-scale production of mAbs, hybridoma cells were adapted to HyClone SFM4MAb-Utility media (GE Healthcare) and grown in a CELLine Classic CL1000 bioreactor (Argos Technologies) according to the manufacturer’s instructions. mAbs were harvested once per week (or when cell density in the bioreactor reached > 4 × 10^8^ cells/mL) and purified under endotoxin-free conditions using HiTrap MabSelect SuRe columns (GE Healthcare) and standard affinity chromatography methods using an ÄKTA system (GE Healthcare). Eluted fractions containing mAbs were pooled and dialyzed against endotoxin-free Dulbecco’s modified phosphate buffered saline (DPBS) pH 7.6 at 4 °C using Slide-A-Lyzer™ Dialysis Cassettes, then aliquoted and stored at − 80 °C until use. The endotoxin concentration of antibody batches was measured using a kinetic chromogenic endpoint assay (Limulus amebocyte lysate; PharmaControl). The mAbs used for in vivo injections had endotoxin levels < 1.0 EU/mL [26].

### Control antibodies

A polyclonal IgG-mix (purified as above) from non-Tg C57Bl/6 mouse serum was used as a control in studies where both IgG2b (αSOD1^65–72^) and IgG1 (αSOD1^143–153^) serotype mAbs were used. A mouse anti-c-MYC IgG1 mAb (clone 9E10) was used as an isotype control for the study in aged mice using only IgG1 (αSOD1^143–153^). 9E10 was developed by Bishop, J.M. and obtained from the Developmental Studies Hybridoma Bank, created by the NICHD of the NIH and maintained at The University of Iowa, Department of Biology, Iowa City, IA 52242.

### Enzyme-linked immunoassays

The reactivity of mAbs against hSOD1 was ascertained by screening serial dilutions of hybridoma culture media, purified mAbs, serum and cerebrospinal fluid (CSF) from immunized mice using an ELISA to disordered hSOD1 (SOD1 ELISA). Briefly, ELISA plates (Maxisorp, Nunc A/S) were coated overnight at 4 °C with 0.3 μg/mL of disordered hSOD1 that had been denatured with guanidinium chloride and metals removed with diethylenetriaminepentaacetic acid [15]. After washing and blocking, antibody solutions were incubated at 37 °C for 90 min. Following washing, a peroxidase-labelled anti-mouse secondary antibody (DAKO) was incubated for 1 h, followed by washing and development in substrate (1,2-phenylenediamine and H_2_O_2_). Absorbance was measured at 490 nm and normalized to blank wells without hSOD1 coating. Mapping of anti-SOD1 antibody binding epitope was conducted using ELISA against partially overlapping synthetic peptides (1 μg/mL) corresponding to the aa 57–72 or 131–153 regions of hSOD1.

### Surface plasmon resonance

Antibodies were immobilized at a density of 10,000–15,000 response units (RU) on a CM5 chip (GE Healthcare) using standard amine-coupling chemistry at pH 5. Denatured hSOD1 was used to determine the monomeric affinity constant of each mAb at a flow rate of 100 μL/min at 25 °C in PBS (pH 7.4). SPR sensorgrams were corrected for non-specific interactions to a reference surface and by double referencing. Experiments were performed using a ProteOn XPR36 system with ProteOn Manage software (Bio-Rad) to calculate k_a_ and k_d_ by selecting the Langmuir (1:1) kinetic fit model.

### Immunocapture

Antibodies were coupled to Dynabeads M-280 Tosyl activated at 20 μg/mg according to the manufacturer’s instructions. Beads were recovered with a magnet and incubated with 0.05% (w/v) BSA, before washing with PBS containing 0.5% (v/v) Nonidet P-40 (NP-40) to remove unbound antibodies. Antibody-coated beads (5 μg antibody) were incubated with 10 μg native hSOD1 or denatured hSOD1 in PBS (pH 7.0) for 1 h at 23 °C. Following washes, immunocaptured hSOD1 was released by boiling the beads in SDS-PAGE sample buffer followed by western blot analysis.

### Mice

Mice were housed on a 12 h light/dark cycle with ad libitum access to food and water. Mice of both sexes were used for experiments. Hemizygous hSOD1^G85R^ (line 148), or hSOD1^G93A^ (G1) [10, 19] Tg mice were used in this study. In our colony, the average lifespan of hSOD1^G85R^ mice is 406 ± 34 days (n = 44) and hSOD1^G93A^ mice 155 ± 9 (n = 170), with no difference in lifespan between male and female mice within each line. The lines have been backcrossed > 30 generations and maintained on the C57BL/6J background. We used age-matched, non-Tg C57BL/6J mice and SOD1 KO mice [33] as controls for western blotting and immunohistochemistry. Unless otherwise stated, mice were sacrificed when considered terminally ill. The criterion for terminal ALS-like disease was defined as almost or full paralysis in both hind limbs and more than 20% loss of body weight. In multiple aged mice, symptoms in forelimbs were more prominent. In these cases, the end stage was set to when forelimbs showed severe weakness resulting in difficulties to eat wet food, or the presence of an eye infection. 9 mice had to be excluded from the study: 3 mice died during anaesthesia, 2 mice were sacrificed due to surgery related wounds, 3 mice (belonging to three different experimental groups) were terminated before reaching end stage disease and excluded due to abnormal growth or behaviour; (1 mouse tremor; 1 mouse growth retardation, and 1 mouse excessive scratching and hair loss). Finally, 1 mouse from the seed inoculated no-mAb control group (n = 9) was excluded as a single outlier (> 2 StD from mean survival).

### Tissue homogenization

Dissected mouse spinal cord samples were homogenized in 25 volumes of ice-cold PBS containing the anti-proteolytic cocktail Complete (Roche Diagnostics), using an Ultraturrax apparatus (IKA, Staufen, Germany) for 30 s followed by sonication for 1 min. Crude homogenates were analysed by western blotting and dot blotting.

### Western blotting

Crude homogenates were diluted 1:10 in 1 × SDS sample buffer without prior centrifugation and heated for 10 min at 100 °C before separation on Any kD Criterion TGX precast gels (Bio-Rad). Either anti-hSOD1 mAbs (1 μg/mL), mouse anti-c-MYC IgG1 (1 μg/mL) or mouse anti-β-actin (1:100,000; Merck Millipore) were used as primary antibodies and HRP-conjugated anti-mouse IgG (1:10,000, DAKO) as a secondary antibody. Immunocaptured samples were analysed using rabbit polyclonal antibodies generated against a peptide corresponding to amino acids a.a. 24–39 in the hSOD1 sequence [15] and an anti-rabbit IgG (1:10,000, DAKO) as secondary antibody. The immunoreaction signal was visualized using ECL Select reagent (GE Healthcare), recorded on a ChemiDoc Touch Imaging system and analysed using Image Lab software (Bio-Rad).

### Dot blotting and binary epitope mapping

Crude tissue homogenates were further diluted in 20 volumes ice cold PBS supplemented with 1 mM DTT, 1.8 mM EDTA and 1% NP-40 and sonicated again on ice. Homogenates were then centrifuged at 1000×*g* for 10 min at 4 °C and supernatants were serially diluted 1:1 in PBS. Aggregates were captured on 0.2 μm cellulose acetate filters using a 96-well dot blot system (Whatmann GmbH). After washing and blocking with 5% (w/v) milk in Tris buffered saline (TBS) containing 0.1% Tween 20, blots were incubated with anti-hSOD1 antibodies overnight at 4 °C. HRP-conjugated anti-rabbit IgG or anti-mouse IgG antibodies (1:10,000, DAKO) were used as secondary antibodies and the blots were developed as described for western blotting.

We used homogenates of cervical spinal cords from mice perfused transcardially with PBS for dot blot assay of in vivo mAb labelling of hSOD1 aggregates. HRP-conjugated anti-mouse IgG (1:10,000, DAKO) was used to detect binding of mouse IgG. Aggregate load was visualized using an anti-hSOD1 aa 57–72 polyclonal rbAb.

For profiling of hSOD1 aggregate structures, we used BEM. A detailed description of the background to the assay and the protocol is described by Bergh et al. [6]. The protocol is based on eight rbAbs raised against short peptides that cover over 90% of the 153 aa hSOD1 protein (Fig. 1a). In the assay the reaction of the antibodies with aggregates captured on dot blot filters is determined. There is no immunoreactivity to the ordered core of protein aggregates/fibrils or to segments otherwise hidden. Only sequence elements that have lost native contacts and are disordered are detected.

For the BEM assay, 96-well dot blot filters were cut into strips, incubated with 8 hSOD1 peptide antibodies and developed as described above. To allow comparison between filters, a spinal cord homogenate from an end-stage hSOD1^G93A^ Tg mouse was designated as a standard and stained with the rbAb α-SOD1^57–72^. To facilitate comparison of staining patterns, staining intensities of the 8 antibodies with individual homogenates were normalized against the staining obtained with rbAb α-SOD1^57–72^ (set as 100%).

### Preparation of hSOD1 aggregate seeds by centrifugation through a density cushion

Strain A hSOD1 aggregate seeds were prepared from a pool of spinal cords from end-stage hSOD1^G85R^ Tg mice as described previously [7, 8]. Briefly, the protocol involved homogenization in 25 volumes of PBS containing 1% NP-40 and 0.5 M guanidinium hydrochloride using an Ultraturrax and sonication. After clearing by 1000×*g* centrifugation, the homogenate was diluted with 3.33 volumes of water with 1% NP-40 to achieve physiological ionic strength. It was then sonicated again and layered on top of a 4 cm high 13% iohexol cushion (δ = 1.074) and centrifuged at 175,000×*g* for 1 h. Under these conditions, proteinaceous components with a molecular mass > ~5 × 10^6^ Da will be sedimented into the pellet [36]. Therefore, seeds will not contain monomeric, dimeric or any oligomeric hSOD1 species. The pellet was suspended by sonication in a small volume of PBS and stored in aliquots at − 80 °C. The hSOD1^G85R^ strain A seeds contained ~ 1 ng/μl hSOD1, which was estimated by western blotting with a hSOD1 protein standard as previously described, and the total protein was estimated to 220 ng/μl using BCA protein assay.

### Inoculation of hSOD1^G85R^ aggregate seeds into lumbar spinal cord

The seed inoculation protocol has been previously described in Bidhendi et al. [7]. Briefly, ~ 100-day old hSOD1^G85R^ Tg mice (see Table 1, Additional file 1: Tables S2 and S3 for age and number of mice for each group) were anesthetized with 4.5% isoflurane (Baxter) and fixed on a small animal stereotactic frame (Kopf Instruments). Anaesthesia was maintained with 1.5–2% isoflurane using a facemask. Before starting the surgery, carprofen 5 ng/g bodyweight was injected subcutaneously. The back skin above the lumbar spinal cord was opened with a sagittal incision and two small bilateral pockets cut close to the spine to insert spine stabilizing clamps. The muscles above the spine were cut transversally and the meninges punctured with a fine needle. We inoculated 1 μL hSOD1^G85R^ aggregate seed suspension into the left side of the lumbar ventral horn between two vertebrae at the L2-L3 level. In the pilot in vivo study, the inoculated hSOD1^G85R^ aggregate preparation was pre-incubated with αSOD1^143–153^ for 15 min on ice and then stored at − 80 °C until inoculation. The concentration of mAb-SOD1 was adjusted to achieve an estimated 1:1 or 1:10 molar ratio of monomeric SOD1 to antibody. We used a digital display console with 10 μm resolution (model 940-B, Kopf) to facilitate precision needle insertion. The injection velocity was 0.125 μL/min facilitated by an infusion pump (Legato 130, KD Scientific). The syringe was slowly withdrawn, the fascia sutured, and the skin closed using surgical clips and Tissue adhesive (3 M Vetbond). Physiological NaCl were injected subcutaneously to prevent possible dehydration before the mouse was placed back in a clean cage. All mice were carefully observed, documented and weighed on a weekly basis until symptom onset, through disease progression and to end stage paresis. When mice developed muscle weakness and had difficulties to climb, wet food was provided on the cage floor and exchanged once daily.

### Intraperitoneal injections and in vivo pharmacokinetics

To assess pharmacokinetic parameters, 127 ± 5-day old hSOD1^G85R^ mice (n = 3/antibody) were injected i.p. with a single dose of 50 mg/kg of αSOD1^143–153^ or αSOD1^65–72^. Submandibular bleedings were collected at 24 h, and at 7, 14, 21, and 28 days post injection. Anti-SOD1 antibody titre in plasma was analysed by SOD1 ELISA.

*Seed inoculated groups:* hSOD1^G85R^ Tg mice were injected i.p. with αSOD1^143–153^ (10 mg/kg and 50 mg/kg), αSOD1^65–72^ (10 mg/kg and 50 mg/kg) or control IgG (50 mg/kg) once per week for 3 weeks prior to inoculation of hSOD1^G85R^ aggregate seeds. All mice received the fourth antibody injection 24–28 h before surgery. Plasma samples were collected by submandibular bleeding 2–6 h before, and on days 28 and 56 after aggregate inoculation and used to assess circulating antibody titre by denatured hSOD1 ELISA. Mice were monitored as described above and mAb and IgG injections were administered once per week until the mice reached terminal disease stage.

*Non*-*inoculated groups:* Once per week mAb injections were started at 250 days of age using αSOD1^143–153^ (50 mg/kg), control IgG1 (50 mg/kg), or vehicle control (endotoxin free DPBS). Injections were continued until animals reached terminal disease stage.

### Sample collection

Mice inoculated with hSOD1^G85R^ aggregates and treated with antibodies were anesthetized with 4.5% isoflurane to collect CSF from the cisterna magna as described previously [24]. Briefly, mice were fixed in the small animal stereotactic frame and anaesthesia was maintained with 1.5–2% isoflurane using the facemask. The back of the head was swabbed with 70% ethanol and a sagittal incision of the skin was made below the occipital bone. The subcutaneous tissue and muscles were separated at the midline and separated with micro-retractors. The body of the mouse was lowered to achieve an angle of ≈ 135° from the horizontal plane of the fixed head. At this angle, the cisterna magna was visible. The dura mater was punctured using a glass capillary with a 0.5 mm outer diameter and 3–5 μl was collected by capillary force. CSF samples were ejected into screw cap tubes and snap frozen using liquid nitrogen. Samples were stored at − 80 °C until analysis of mAb titre by ELISA. Mice were maintained under anaesthesia with isoflurane and injected with 0.25 mL pentobarbital (60 mg/mL, APL Pharma) for deep terminal anaesthesia. Antibody-treated aged mice and mice inoculated with a pre-mix of antibody and aggregates were directly anesthetized with 0.25 mL pentobarbital (60 mg/mL). After blood collection from the vena cava, animals were perfused transcardially with 50 mL PBS (5 mL/min). Following decapitation, the brain was dissected and divided into left and right hemispheres, brain stem and cerebellum. The spinal cord was extracted by flushing the vertebral column from the caudal end with a syringe containing PBS and divided sagittally into left and right sides, and then into cervical, thoracic and lumbar segments. Tissues from the left side were snap-frozen in liquid nitrogen and stored at − 80 °C for biochemical analyses. Tissues from the right side were fixed in formalin and paraffin embedded for immunohistochemistry.

### Immunohistochemistry

Paraffin embedded spinal cord tissue was cut into 4–6 μm thick sections from individual tissue blocks or tissue microarray blocks generated with the TMA Grand Master (3DHISTECH). Blocks were cut with a standard rotary microtome (Microm HM400) and sections were processed simultaneously under the same conditions.

For immunoperoxidase staining, tissue sections were processed with the automated BenchMark Ultra (Ventana Medical System Inc.) according to the manufacturer’s instructions. Following deparaffinization at 72 °C, sections were either incubated directly with mAbs without antigen retrieval treatment, to visualize monomeric disordered and aggregates of SOD1, or pre-treated with Ventana Protease 1 solution for 4 min to clear the tissue from monomeric denatured, or disordered SOD1, to visualize large aggregated SOD1 species. Sections were then incubated for 32 min with the mAb 5.7 (2 μg/mL and 3 μg/mL), mAb 134.2 (αSOD1^65–72^, 6 μg/mL and 0.6 μg/mL), mAb 85.11 (αSOD1^143–153^, 5.7 μg/mL and 1 μg/mL) and mAb 545.2 (0.3 μg/mL). Next, tissues were incubated with horseradish peroxidase (HRP)-conjugated secondary antibody and immunoreactions visualized using the *iView* Universal DAB detection kit (Ventana). After counterstaining with hematoxylin, sections were de-hydrated and mounted with Pertex (Histolab). Slides were scanned with the Pannoramic Digital Slide Scanner (3DHISTECH) and images used for analysis were acquired with CaseViewer software (3DHISTECH).

Sections stained with the primary antibodies anti-Iba1 (1:1000, rabbit, microglia marker, WAKO), anti-GFAP (1:5000, rabbit, astrocyte marker, DAKO) or anti-ChAT (1:750, rabbit, cholinergic neuron marker, including MNs, Merck Millipore) were pre-treated with Cell Conditioning 1 (CC1, pH 6) solution at 95 °C for 36 min (Iba1, GFAP) or 64 min (ChAT). Tissues were then incubated with primary antibodies for 32 min, secondary antibody and visualized as described above.

For immunofluorescence staining, sections were deparaffinized, rehydrated and antigens retrieved in 10 mM citrate buffer (pH 6.0) using a pressure cooker (2100 Antigen Retriever, Aptum Biologics). After blocking with 10% (v/v) FBS in PBS containing 0.3% (v/v) Triton X-100, sections were incubated overnight at 4 °C with primary antibodies anti-Iba1 (1:1000), anti-GFAP (1:500), anti-hSOD1 against amino acids 131–153 (1:1000, chicken, in house) for 48 h at 4 °C with anti-ChAT (1:200, goat, Millipore) simultaneously with anti-hSOD1 against amino acids 131–153 (1:1000, rabbit, in house) and biotinylated anti-mouse IgG (1:500, horse, Vector Laboratories). Sections were washed with PBS and incubated with Alexa-Fluor conjugated secondary antibodies (1:1000) and 4′,6-diamidino-2-phenylindole (DAPI; 0.3 μM) for nuclear counterstaining. Following washing with PBS, sections were mounted with Fluorescence Mounting Media (Dako). Fluorescence images were obtained with a Nikon A1 confocal microscope using a 40× water immersion lens (N.A. 1.2) or scanned with a Vectra 3.0 Automated Imaging System (PerkinElmer). All images were processed and analysed with ImageJ software.

### Statistical analyses

Statistical analyses were performed using Prism 6.00 (GraphPad). Mann–Whitney U test was used to test for statistical significance between two groups. To test for statistical significance between multiple groups, we used the Kruskal–Wallis test followed by Dunn’s post hoc test. The significance level was set to < 0.05. All values are given as means ± SD.

## Supplementary information

**Additional file 1.** mAb characterization data, disease and survival data, and characterization of spinal cord tissue from mAb treated mice.

## Data Availability

The authors declare that the data supporting the findings of this study are available within the article or its supplementary information file.
