# Supplementary material for: Aggregate-selective antibody attenuates seeded aggregation but not spontaneously evolving disease in SOD1 ALS model mice
Source: Acta Neuropathol Commun. 2020 Sep 14;8:161. doi: 10.1186/s40478-020-01032-2 (PMC7488686; doi:10.1186/s40478-020-01032-2)
Supplement: Supplementary file 1 — Additional file 1. mAb characterization data, disease and survival data, and characterization of spinal cord tissue from mAb treated mice. [file 40478_2020_1032_MOESM1_ESM.pdf]

Supplementary material

Aggregate-selective antibody attenuates seeded aggregation but not spontaneously evolving disease in SOD1 ALS model mice

Manuela Lehmann[1], Matthew Marklund[2], Anna-Lena Bolender[1], Elaheh E. Bidhendi[1], Per Zetterström[3], Peter M. Andersen[1], Thomas Brännström[2], Stefan L. Marklund[3], Jonathan D. Gilthorpe[4], Ulrika Nordström[1*]

[1] Department of Clinical Science, Neurosciences, Umeå University, Umeå 901 87, Sweden.
[2] Department of Medical Biosciences, Pathology, Umeå University, Umeå 901 87, Sweden.
[3] Department of Medical Biosciences, Clinical Chemistry, Umeå University, Umeå 901 87, Sweden.
[4] Department of Integrative Medical Biology, Umeå University, Umeå 901 87, Sweden.

* To whom correspondence should be addressed. Email: ulrika.nordstrom@umu.se, +46-73 7396695

Supplementary Tables

SOD1 peptide	Disordered / Denatured hSOD1									Aggregated hSOD1			Native hSOD1
	mAb clone	Isotype	Denat hSOD1 ELISA	SPR			WB	Immuno-capture	IHC	Dot blot (Strain A)	Complex Stability	IHC	Immuno-capture
				k_a	k_d	KD							
57-72	**134.2***	**IgG2b**	**100**	**4.6**	**2**	**0.4**	**+++**	**+++**	**+++**	**100**	**++**	**++**	–
	**5.7**	**IgG1**	**101**	**2**	**5.6**	**2.7**	**+++**	**+++**	**+**	**119**	**++**	**++**	–
	27.7	IgG1	71	0.9	14.2	16	n.a.	n.a.	n.a.	163	n.a.	n.a.	n.a.
	62.5	IgG2a	19	0.1	1.3	10	n.a.	n.a.	n.a.	79	n.a.	n.a.	n.a.
131-153	**85.11***	**IgG1**	**100**	**1.7**	**21.3**	**12.5**	**++**	**++**	**+++**	**256**	**+++**	**+++**	–
	**545.2**	**IgG2b**	**29**	–	–	–	**++**	**+**	**+++**	**295**	**+**	**+++**	–
	172.16	IgG1	36	–	–	–	n.a.	n.a.	n.a.	65	n.a.	n.a.	n.a.
	457.3	IgG1	42	–	–	–	n.a.	n.a.	n.a.	138	n.a.	n.a.	n.a.
	30.5	IgG2b	44	–	–	–	n.a.	n.a.	n.a.	102	n.a.	n.a.	n.a.

Table S1. Summary of *in vitro* characterization of the αSOD1 peptide mAbs

Nine mAb clones that displayed reactivity towards denatured hSOD1 in an ELISA assay were evaluated further with regard to their affinity for disordered and aggregated hSOD1 from terminal stage hSOD1 Tg mouse spinal cord homogenates. Based on low dissociation constant and high reactivity with aggregates in the dot blot assay, two mAbs raised against the aa 57-72 peptide, and two mAbs raised against the aa 131-153 peptide were selected for further characterization. (**hSOD1 ELISA**) Denatured hSOD1 ELISA. Values relative to mAb clone 134.2 ($\alpha SOD1^{65\text{-}72}$) binding set as 100%. (**SPR**) Surface Plasmon Resonance. (Ka) association rate constant ($^{x}10^{4}$ $M^{-1}s^{-1}$), (Kd) dissociation rate constants ($^{x}10^{-4}$ s^{-1}) and (KD) binding constants (nM). (**WB**) Western blot (**i.c.**) Immunocapture. (**IHC**) Immunohistochemical staining of tissue sections. (1) Tissue sections treated with antigen retrieval pH 6.0. (2) Tissue sections treated with proteinase to degrade disordered monomeric and oligomeric SOD1 species and stain preferentially proteinase resistant aggregated hSOD1 species. (**Dot blot**) Immunoblotting of filter-trapped hSOD1 aggregates isolated from end-stage $hSOD1^{G93A}$ Tg mouse spinal cord. Values are presented as relative to mAb clone 134.2 ($\alpha SOD1^{65\text{-}72}$) binding, set as 100%.

	hSOD1^{G85R} seed + vehicle		hSOD1^{G85R} seed + αSOD1$^{143-153}$ (1:1)		hSOD1^{G85R} seed + αSOD1$^{143-153}$ (1:10)	
	n	days	n	days	n	days
Age at seed-inoculation	6	107 ± 3	6	108 ± 2	6	108 ± 2
Symptom onset (days post-inoculation)	6	86 ± 26	5	122 ± 40	5	113 ± 39
Paralytic end-stage (days post-inoculation)	6	92 ± 22	6	160 ± 76	6	154 ± 77
Age at paralytic end-stage	6	199 ± 24	6	268 ± 77	6	263 ± 77
Duration of disease	6	13 ± 3	5	11 ± 2	5	15 ± 8

Table S2. Summary of ALS-like disease progression after inoculation of G85R aggregate seeds
All data are presented as mean ± SD. Time between inoculation of seed preparations pre-mixed with αSOD1$^{143-153}$ and end-stage ALS-like disease was essentially equal for both groups. Analyzed as one group, mice inoculated with seeds mixed with αSOD1$^{143-153}$ had a significantly longer post-inoculation survival time compared to control mice inoculated with seeds mixed with vehicle. * $p < 0.05$.
n = number of animals.

	vehicle		IgG1		αSOD1$^{143-153}$	
	n	days	n	days	n	days
1st mAb injection	10	254 ± 1	9	253 ± 4	11	253 ± 3
Symptom onset	9	369 ± 36	8	394 ± 42	11	377 ± 40
Terminal stage	10	395 ± 43	9	403 ± 41	11	390 ± 42
Duration of disease	9	18 ± 7	8	15 ± 5	11	13 ± 7

Table S3. Symptom and survival data of vehicle or mAb treated non-inoculated hSOD1^{G85R} Tg mice
All data are presented as mean ± SD.
There was no significant difference in survival between mAb treated mice and vehicle treated mice.

Supplementary figure legends

Figure S1. αSOD1 mAbs show selective reactivity to SOD1 in mouse spinal cord homogenates and tissue sections

(a) Image shows a full view of a denaturing western blot (WB) filter used for mAb binding analyses (c.f. Fig. 1e). Crude spinal cord homogenates were isolated from SOD1 knockout (KO), C57BL/6J non-Tg control (Wt) and hSOD1^{G85R} Tg mice and probed with control and αSOD1 mAbs. All mAbs react with a ~17 kDa SOD1 band and show no, or only weak, cross-reactivity. Control antibodies (mAb IgG1, polyclonal mouse IgG) show no reactivity to mouse or human SOD1. **(b)** Images show DAB immunostaining of spinal cord paraffin sections from SOD1 KO, non-Tg C57BL/6J control (Wt), and hSOD1^{G85R} Tg mice; pre-symptomatic (200-day), and (400-day) terminal ALS-like disease stage. Distinct staining in the ventral horn of Tg mice, but not in SOD1 KO confirm specific mAb antigen reactivity in tissue sections. Scale bar = 40 μm.

Figure S2. SOD1 levels and aggregate structural conformation are unaltered in the CNS of mAb treated mice

(a) Graph depicts circulating antibody concentrations used for calculating mAb elimination rate in plasma samples from C57BL/6J mice injected with a single dose of 50 mg/kg of αSOD1$^{65\text{-}72}$ or αSOD1$^{143\text{-}153}$, respectively. Blood samples were collected on days 1, 7, 14, 21, and 28 post mAb injection and analysed by SOD1 ELISA. Values represent mean ± SD (n = 3 mice for each mAb). **(b)** Image shows western blot analysis of soluble SOD1 protein in cervical spinal cord homogenates from terminal stage seed inoculated and mAb treated mice, using the human specific rbAb hSOD1$^{24\text{-}39}$ (upper lanes) or murine specific rbAb targeting murine SOD1$^{24\text{-}36}$ sequence (lower lanes). **(c)** Graph shows estimated levels of soluble hSOD1 in (B). Values represent relative intensities of bands in western blots (mean ± SD) and were normalized to total protein (n = 3 mice/group). **(d)** Graph depicts estimated relative aggregate load in cervical spinal cord homogenates analysed by dot blotting using rbAb αSOD1$^{57\text{-}72}$. Values represent relative intensities (mean ± SD) (n = 3 mice/group). **(e)** Binary epitope-mapping (BEM) patterns of hSOD1 aggregates extracted from terminally ill mice; without mAb treatment (black); treated with 50 mg/kg αSOD1$^{143\text{-}153}$ (red) and 50 mg/kg αSOD1$^{65\text{-}72}$ (green) (n = 3 mice/group). The *x*-axes represent the hSOD1 peptide sequences

targeted by the eight polyclonal αSOD1 rbAbs used in the BEM assay. Binding of the respective rbAb is shown as relative intensity, normalized to rbAb 57-72 intensity, set as 100%.

Figure S3. Analysis of mAb labelling in association to hSOD1 aggregation, ChAT$^+$ motor neurons and Iba1$^+$ microglia

(a,b) Images represent immunofluorescence labelling to assess mAb/hSOD1 aggregate association in cervical spinal cord tissue sections from terminal seed inoculated and αSOD1 treated terminal hSOD1^{G85R} Tg mice. Anti-mouse IgG was used to label mAbs. **(a)** Anti-mouse IgG immunofluorescence was detected in association to hSOD1 aggregates in αSOD1$^{143\text{-}154}$ treated mice. No clear hSOD1 aggregate/mAb association was detected in tissue sections from αSOD1$^{65\text{-}72}$ treated animals. Only microvessel-like structures show anti-mouse IgG labelling in these mice. Scale bar = 20 µm. **(b)** Image shows mouse IgG (m-IgG) labelled hSOD1 aggregate assembly surrounded by an Iba1$^+$ microglial rosette. Similar microglial rosettes were found around non-mAb labelled aggregates in the αSOD1$^{65\text{-}72}$ treated mice. Scale bar = 10 µm. **(c,d)** Graphs show number of ChAT$^+$ motor neurons: **(c)** small 100-300 µm^2 and **(d)** large >300 µm^2 in end-stage mice. There was no significant difference between treatment groups. All data is presented as mean ± SD from (n = 3-5 animals/group, 3-5 sections/mouse).

Figure S4. αSOD1 mAb treatment does not alter accumulation of hSOD1 aggregates in neuroglia

Images show double immunohistochemical labelling of hSOD1 aggregates and **(a)** astrocytes (GFAP$^+$)**,** or **(b)** microglia (Iba1$^+$), in spinal cords of terminal seed inoculated hSOD1^{G85R} Tg mice treated with control IgG mAb, or αSOD1 mAb. Age-matched C57BL/6 Wt control mouse was included for comparison, to visualize the extensive astrogliosis and microgliosis in end-stage hSOD1^{G85R} Tg ALS model mice. Aggregated hSOD1 associated with glia was found in only a few occurrences in all treatment groups. Thus, αSOD1 mAb treatment did not result in increased accumulation of aggregated hSOD1 in astroglia and microglia. Scale bar = 100 µm. **(c)** Potential differences in microglia activation was assessed by quantification of the pixel area of Iba1$^+$ staining in cervical ventral horn sections. Values are expressed as

percentage of $Iba1^+$/total pixel area. All data is presented as mean ± SD (n = 4-7 mice/group, 3 sections/mouse). $Iba1^+$ pixel count was not significantly different between treatment groups.

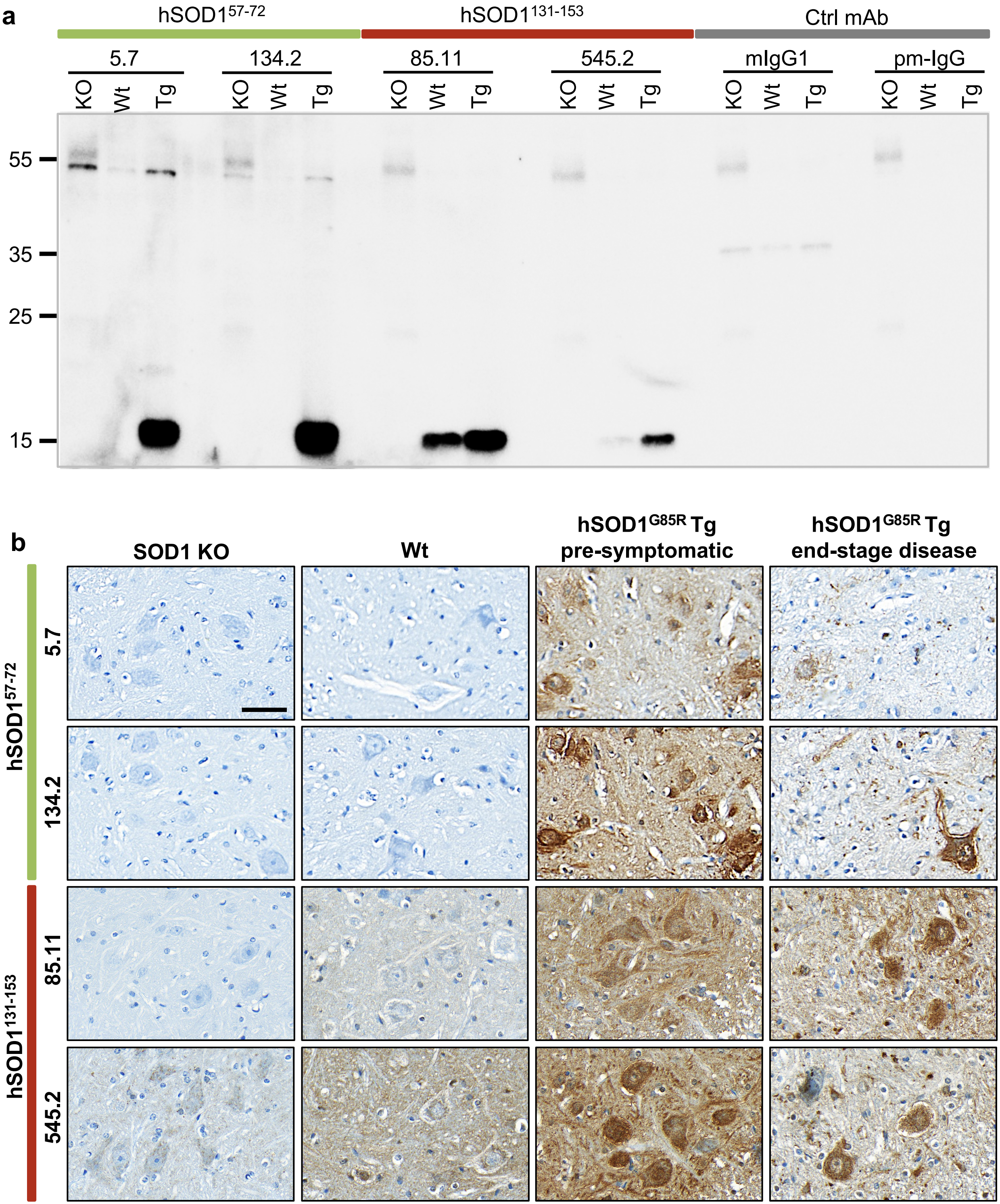

Figure S1. Lehmann et al.

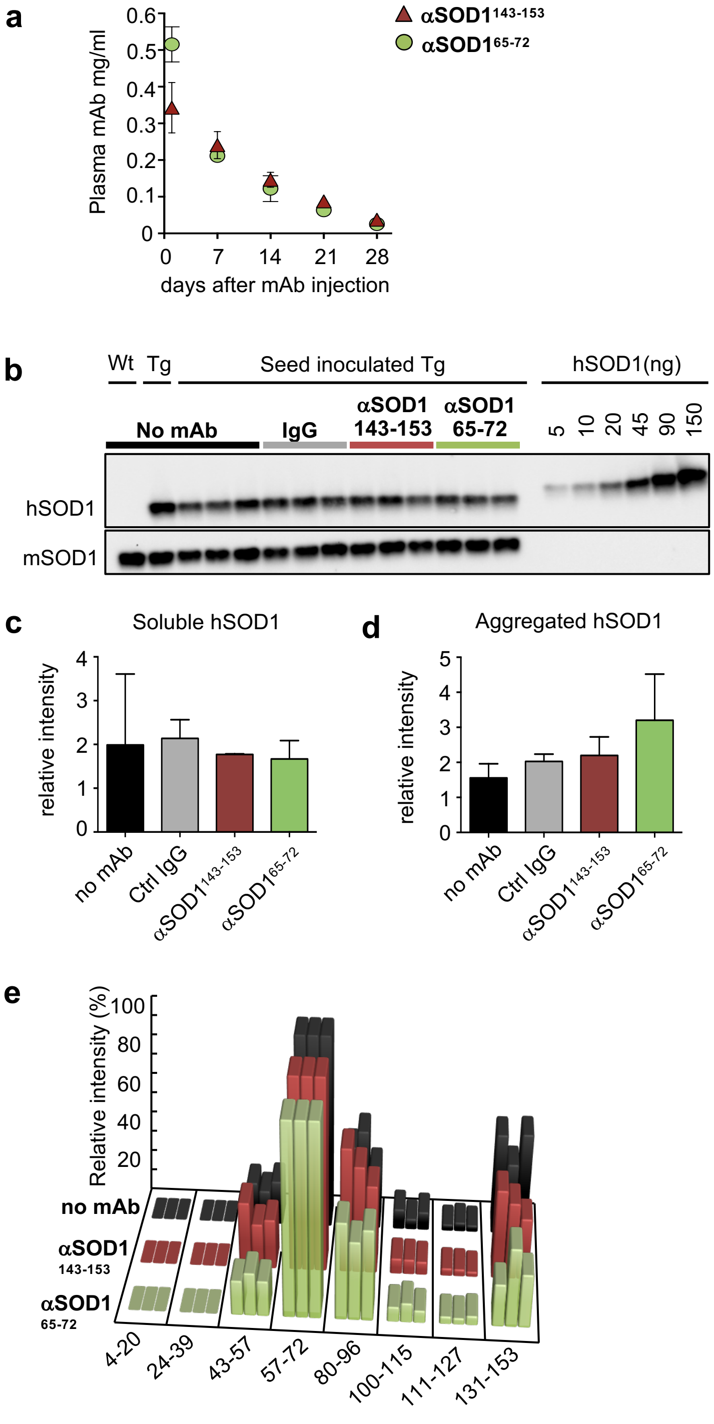

Figure S2. Lehmann et al.

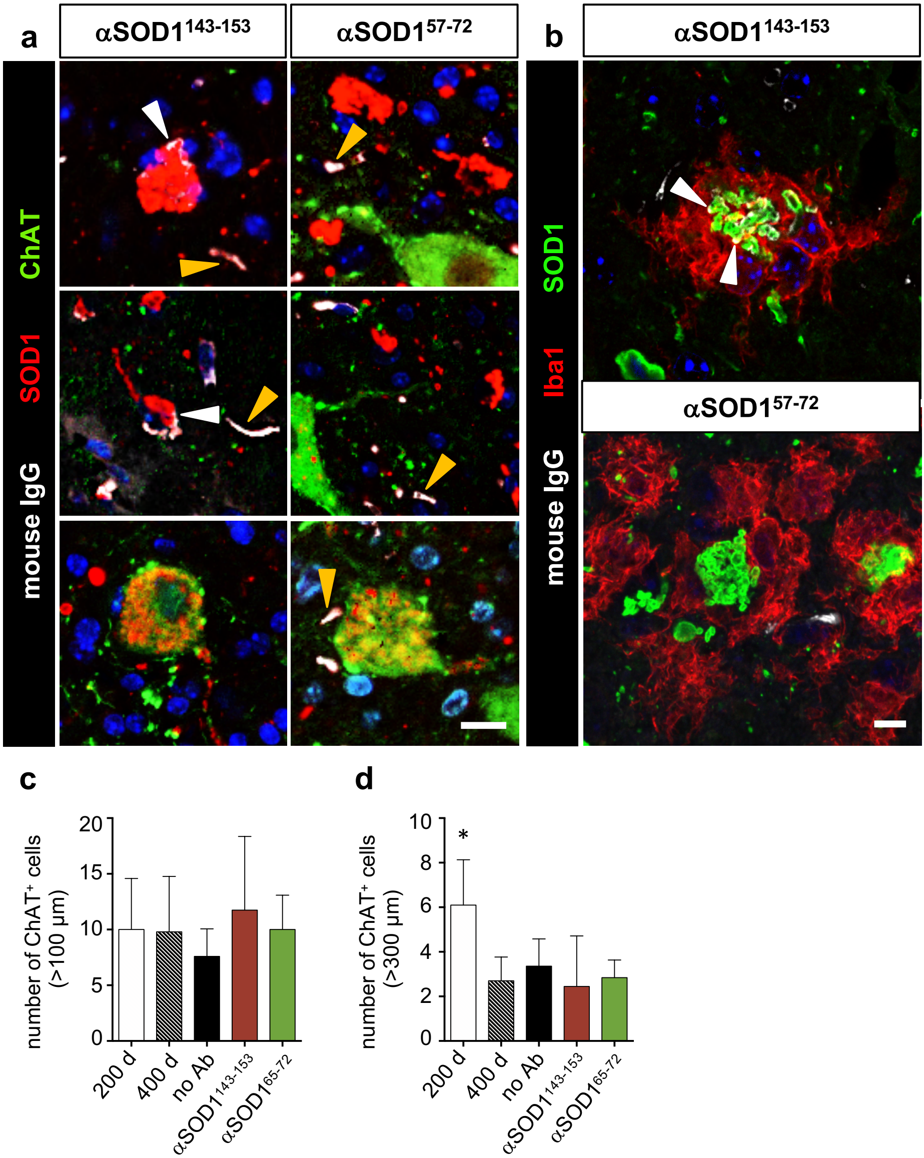

Figure S3. Lehmann et al.

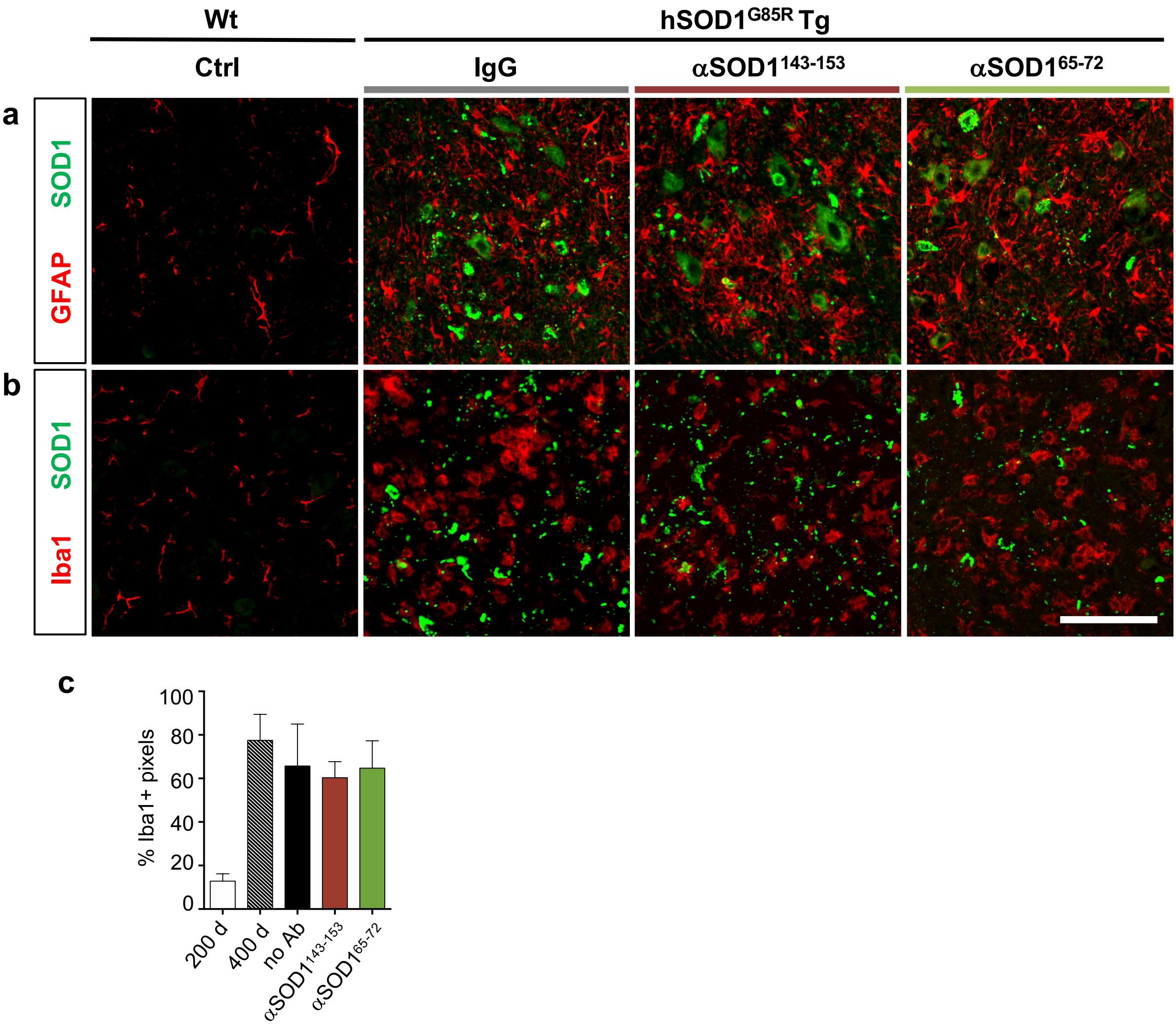

Figure S4. Lehmann et al.
